# Prevalence of BRAF Mutation in Colorectal Cancer Among Lebanese Patients: A Descriptive Study

**DOI:** 10.3390/jcm15051913

**Published:** 2026-03-03

**Authors:** Christelle Rahme, Bassil Josianne, Trak Smayra Viviane, Kattan Joseph

**Affiliations:** 1 Department of Internal Medicine, College of Medicine, Central Michigan University, Mount Pleasant Campus, Mount Pleasant, MI 48859, USA; 2 Department of Hematology and Medical Oncology, Gustave Roussy Institute, 94800 Villejuif, France; 3 Department of Pathology, Hôtel-Dieu de France, Saint Joseph University of Beirut, Beirut 166830, Lebanon; viviane.traksmayra@usj.edu.lb; 4 Department of Hematology and Medical Oncology, Hôtel-Dieu de France, Saint Joseph University of Beirut, Beirut 166830, Lebanon

**Keywords:** colorectal cancer, BRAF mutation, molecular profiling, prevalence, Lebanese population, epidemiology, gene mutation

## Abstract

**Background**: Although the BRAF gene mutation in colorectal cancer has a prognostic value and a therapeutic interest, very few studies address the prevalence of this mutation in the Middle East, and hardly any among the Lebanese population. Moreover, we studied the correlation between this mutation and other clinical and pathological variables. **Methods**: In this descriptive, retrospective, single-center study, BRAF mutational status was reviewed in colorectal tumor samples collected from 2015 to 2021 of Lebanese patients with confirmed metastatic colorectal cancer. The genetic analysis was done in two different molecular laboratories. Clinical characteristics were selected from the computerized medical records of included patients. Statistical calculations were performed with SPSS (version 21.0) statistical software. **Results**: The study included 190 patients. BRAF mutation was detected in 10 patients (5.3%). A positive correlation was observed between the presence of a BRAF mutation and the right-sidedness of the tumor (*p* = 0.001) as well as with the presence of microsatellite instability (*p* = 0.004). However, we could not establish a relationship between BRAF mutation and other characteristics such as age (*p* = 0.682), gender (*p* = 0.392), the degree of histologic differentiation (*p* = 0.594), and the presence of peritoneal metastases (*p* = 0.707). **Conclusions**: The BRAF mutation was found in 5.3% of colorectal cancers in Lebanon. A positive correlation was suggested with the colon sidedness and the microsatellite instability. However, it was still insufficient to establish statistically significant associations between other variables and the BRAF mutation.

## 1. Introduction

While the incidence of colorectal cancer (CRC) has been increasing over the past decade [1], the molecular alterations underlying its development, as well as corresponding targeted therapies, are becoming well-defined. CRC’s molecular profile includes a wide range of molecular markers; nevertheless, in routine practice, the molecular profile of metastatic CRC is often limited to the determination of the stability of the microsatellites and the mutation of KRAS and NRAS, in addition to BRAF [2]. The serine/threonine protein kinase B-raf (BRAF) is a mutated oncogene found in various neoplasms, including melanoma and CRC. The V600E mutation is the most common BRAF gene mutation in humans. It consists of the substitution of valine for glutamic acid at the 600th codon. This is due to a point mutation that substitutes thymine for adenine at the 1799 position. The result is a constitutive activation of the epidermal growth factor receptor (EGFR) circuit [3]. This circuit allows the transmission of an extracellular message to the nucleus of the cell targeting the genes responsible for activating cell proliferation, division, and differentiation. BRAF, as a pathway signaling protein, is preceded by RAS, a small guanosine triphosphatase (GTPase), and followed by the Mitogen-activated protein kinase kinase (MEK) protein, which eventually leads to the phosphorylation of multiple transcription factors [4]. BRAF-mutated CRC tumors have been associated with a peculiar phenotype. Tumors with BRAF V600E mutations are more likely to be seen in older patients and in current or former smokers, particularly white persons and women. It is found at higher frequencies in cancers with proximal location, poor differentiation, mucinous etiology, and microsatellite instability (MSI) [5]. Moreover, BRAF-mutated CRC tumors progress more frequently to T4, and present higher rates of peritoneal involvement and less frequently present liver-limited or lung metastases. Overall, BRAF V600E mutation is associated with poor clinical outcome, especially if it is combined with the mutational status of microsatellites. Combined BRAF/MSI status in colorectal cancer is a more accurate prognostic molecular biomarker, with (MSS/BRAF-mutant) having the poorest prognosis [6]. Moreover, the treatment benefit in BRAF V600E tumors is much lower than BRAF wild-type tumors. For instance, BRAF inhibitors have been shown to be highly effective in melanomas [7], and not in CRC, where the existence of the mutation indicated a rather poor response to these treatments [8]. However, a recent randomized study conducted by S. Kopetz et al. on a triple combination of a MEK inhibitor (Binimetinib), a BRAF V600E inhibitor (Encorafenib), and an anti-EGFR (Cetuximab) in BRAF V600E mutated colorectal cancer showed promising results. A total of 665 patients, who experienced treatment failure with one or two prior regimens, were enrolled. During the study, 224 received the triplet-therapy, 220 received the doublet-therapy Encorafenib and Cetuximab only, and 221 were treated by either Cetuximab and Irinotecan or Cetuximab and FOLFIRI as a control group. The median overall survival was 9.0 months in the triplet-therapy group, 8.4 months in the doublet-therapy group, and 5.4 months in the control group. The overall response rate was 26%, 20%, and 2%, while the median progression-free survival was 4.3, 4.2, and 1.5 months in the three groups, respectively. Adverse events of grade three or higher were observed in 58%, 50%, and in 61% of the patients [9]. With these important prognostic and therapeutic implications of BRAF mutations in the CRC approach, epidemiological studies on BRAF mutation prevalence in a specific geographic region and ethnicity are warranted.

Malignancy represents a significant public health burden in Lebanon. Recent estimates from the Global Burden of Diseases, Injuries, and Risk Factors Study (GBD) 2023 rank Lebanon as having one of the fastest-rising rates of cancer incidence and mortality worldwide between 1990 and 2023 [10]. CRC is among the most prevalent malignancies in the country, with emerging evidence indicating a concerning increase in incidence among individuals younger than 50 years of age. The increasing number of CRC cases has been linked to several factors, including population aging, smoking, alcohol consumption, unhealthy diet, sedentary lifestyle, and environmental pollution [11]. Despite this substantial burden, population-based data on CRC in Lebanon remain scarce, highlighting the need for further studies in this population.

Herein, we report the first study to establish, among other elements, the prevalence of the somatic BRAF mutation through a retrospective descriptive report in patients with colorectal carcinoma in Lebanon.

## 2. Materials and Methods

### 2.1. Study Population

This is a retrospective, descriptive, single-center epidemiologic study that seeks to determine the frequency of the BRAF gene mutation in colorectal cancer Lebanese patients. The study population includes the molecular analysis of samples collected at our pathology department between 2015 and 2021 from patients above 18 years of age, with histologically or cytologically confirmed metastatic colorectal carcinoma. The ethical committee approval at Hôtel-Dieu de France University Hospital was obtained. The collection of the results of the genetic study, as well as the patients’ electronic records (electronic software DxCare version 7.7.9.7.13), provided the data necessary for the characterization of the tumors studied. Clinical characteristics such gender, age, location of the tumor, its sidedness and site of metastases were collected. The pathology records included tissue from primary tumors or metastases that had been surgically resected or biopsied. Grade of the pathology, as well as MSI assessment, was determined by immunohistochemistry.

### 2.2. Genetic Studies

The study of the molecular profile of the samples was done in two distinct genetic study centers. A total of 41 samples were studied at the Medical Genetics Unit (MGU) of the Faculty of Medicine of Saint Joseph University of Beirut (USJ) and 149 samples at BioMarker Solutions Ltd. in Norwich, UK. The samples from the MGU were initially tested only for the KRAS mutation. Should the mutation be missing, the genetic study looked for the molecular profile of 18 mutations in the NRAS gene and five mutations in the BRAF gene. The study of these genes was carried out using a real-time qualitative PCR research technique (Idylla TM). The genetic study BioMarker Solutions Ltd. was performed by extracting DNA from the study sample using the Qiagen QIAmp DNA FFPE tissue kit (QIAGEN N.V., Hilden, Germany). A targeted library was generated using the AmpliSeqTM Cancer Hotspot Panel V2 Ion kit (Thermo Fisher Scientific, Waltham, MA, USA), and analyzed by next-generation semiconductor-based sequencing on Ion Torrent PGM (Thermo Fischer Scientific, Waltham, MA, USA). A total of 25 exons were targeted in the BRAF, NRAS, KRAS, EGFR, PIK3CA, P53, and PTEN genes. For the MSI analysis, DNA was extracted from the test sample using the Qiagen QIAmp DNA FFPE tissue kit. DNA was extracted from both invasive neoplastic tumor cells and normal (or alternative) surrounding tissue as a control for analysis. The stability of the microsatellites was also investigated, but only at the request of the treating physician. The analysis was performed in a laboratory equipped with ISO 15189 [12].

### 2.3. Statistical Analysis

Several variables were added to the statistical analysis to study the interdependence between various parameters. Statistical calculations were performed using IBM SPSS Statistics for Windows, Version 21.0. Armonk, NY, USA: IBM Corp. For the majority of the variables, Pearson’s chi-squared test (χ^2^ test) was used to analyze the results. The dependency of BRAF mutation and age was studied using Student’s *t*-test. The significance level was set at *p* < 0.05

## 3. Results

### 3.1. Characteristics of the Population

A total of 190 cases during a seven-year period (2015–2021) were included. There were 101 (53.2%) men with an average age of 62, and 89 (46.8%) women with an average age of 61. The BRAF mutational status was studied in all 190 specimens. MSI testing was performed on 87 (45.8%) tissue specimens, revealing four (4.6%) specimens with labeled MSI. Among the enrolled patients, 70 (61.4%) patients had peritoneal metastases while 44 (38.6%) had metastases in other locations, mainly hepatic, pulmonary, or bone metastasis. Regarding the sidedness of the tumor, 91 (48%) patients presented with tumors on the left side and 40 (21%) on the right side. The most common sites in descending order of frequency are as follows: rectum, sigmoid, cecum, transverse colon, left colonic angle, recto-sigmoid, and right colonic angle. Finally, the grade of differentiation in the histological report was evaluated in 106 (55.8%) patients and shows that the majority (88 = 83.0%) of patients present moderately differentiated tumors (Table 1). Among the 190 CRC cases, 10 were found to carry a BRAF V600E mutation. Thus, the calculated prevalence of the BRAF mutation in colorectal cancer in our sample of Lebanese patients was found to be 5.3%.

### 3.2. Relationship Between the BRAF Mutation and Other Studied Parameters

#### 3.2.1. Age

All our patients were over 18 years with a mean age at CRC diagnosis of 61.39 years and a median age of 63 years. The interdependence between age and BRAF mutation was studied using Student *t*-test. The mean age at cancer diagnosis in patients with non-mutated BRAF tumors was found to be 61.44 ± 13.118 years, while in mutated BRAF tumors, it was higher, at 63.38 ± 12.141 years (Table 2). The value of the bilateral asymptotic significance of the calculated *t*-test is *p* = 0.682; however, the result is statistically insignificant.

#### 3.2.2. Gender

In the male population, 4% of colorectal tumors are BRAF-mutated, while that figure increases to 6.7% in women. Among the sample studied, 40% of BRAF mutations are found in men and 60% in women, but the association between the gender and the prevalence of BRAF mutation was not statistically significant since the bilateral asymptotic significance value was 0.392.

#### 3.2.3. Microsatellites

A total of 87 samples were analyzed for microsatellite analysis, 83 (95.4%) of which were MSS, and four (4.6%) were MSI. Eight of the 10 tumors with a mutated BRAF had a microsatellite analysis result, six of which had an MSS profile (75%). A total of 79 of the tumor samples with wild-type BRAF had a microsatellite analysis. A total of 77 had MSS (97.5%), and two had MSI (2.5%) (*p* = 0.004).

#### 3.2.4. Sidedness

A total of 91 (48%) of the CRC tumors were found on the left side, while 40 (21%) were found on the right side. In total, 89 out of 122 (73.0%) of the wild-type BRAF tumors were found on the left, and 33 out of 122 (27.0%) on the right. Two (22.2%) tumors with mutated BRAF were found on the left, and seven (77.8%) of the BRAF-mutated tumors were found on the right. The dependency between these two variables was calculated by the Pearson’s chi-squared test and showed a bilateral asymptotic significance of 0.001 with a 95% safety factor.

#### 3.2.5. Location of the Tumor

The location of the tumor was studied in more detail by dividing the sites into seven anatomical zones. Almost half of the wild-type tumors occurred in the rectum, whereas less than 15% of the BRAF-mutated tumors were found. On the other end of the colon, about half of the BRAF-mutated tumors were found in the coecum, while it was the location of approximately 15% of BRAF wild-type. The calculation of the significance of the bilateral values was done using the Pearson’s chi-squared test and found, with a safety coefficient of 95%, an asymptotic of 0.034.

#### 3.2.6. Sites of the Metastases

Metastasis sites were only noted in 114 tumor samples. As for the rest, neither the presence nor the absence of metastases could be proven due to missing data. The sites of metastases were divided into two groups: peritoneal and other locations. The metastatic samples included 70 peritoneal and 44 samples pertaining to “other” locations. These 70 samples included 65 wild-type BRAF tumors and 5 mutated BRAF tumors. Notably, five (55.5%) of the mutated BRAF tumors showed peritoneal metastases compared to 65 (61.9%) of the wild-type BRAF tumors. By the Pearson’s chi-squared test, the result was non-significant. The bilateral asymptotic significance value was 0.707. Consequently, no relationship can be established between BRAF status and metastasis site predilection in this study

#### 3.2.7. Histological Grade

Eight (8.0%) of wild-type BRAF tumors were well-differentiated, 83 (83.0%) were moderately differentiated, and nine (9.0%) were poorly differentiated, whereas one (16.7%) of the mutated BRAF tumors were well-differentiated, five (83.3%) were moderately differentiated, and none were poorly differentiated. The Pearson’s chi-squared test calculated a bilateral asymptotic significance value of 0.594. On that ground, no relationship can be established between histological grade and BRAF mutation status in this study

## 4. Discussion

In this descriptive epidemiologic study, the primary focus was to establish the prevalence of the BRAF mutation in Lebanese CRC patients. Secondary objectives included the examination of potential associations between the BRAF genotype and some clinicopathological phenotypes. To the best of our knowledge, this is the only report to provide data on the frequency of BRAF mutations in an adequate sample size of CRC among the Lebanese population and one of only a few in the Middle East.

The other Lebanese study, conducted by Ibrahim et al., comprised a population of 640 patients. Even so, the search for the BRAF mutation was actually restricted to only 31 patients with metastatic colorectal tumors [12]. In fact, most studies conducted in the region have small sample sizes, and the calculated prevalence of the BRAF mutation in the various Middle East studies shows widely varying values (Table 3). This difference in frequency may be due to the use of different methods/doses to assess the mutation. Nevertheless, the average prevalence across these studies is estimated at 4.56%, with a standard deviation of ± 6.72 [12,13,14,15,16,17,18,19,20,21,22,23]. In our cohort, the prevalence of the BRAF V600E mutation was found to be 5.3%. This value is comparable to those reported in our region but lower than the prevalence observed in the EURO, WPRO, and PAHO regions (8.07%, 5.38%, and 5.55%, respectively) [24]. It is expected that ethnic origin as well as genetic and environmental factors are the factors most likely responsible for the observed differences between different populations around the world.

Clinicopathological characteristics specific to the BRAF mutation have been described in the literature [5,6]. Some of these characteristics have been observed in our population, while the relationship of others to the mutation could not be established. Our study did not show a statistically significant association between the presence of the BRAF mutation and gender. This can be explained by the small number of CRC patients with mutated BRAF (only ten patients), which was insufficient to establish a dependent relationship between the two variables. Similarly, the hypothesis of an association between the mutated BRAF profile and the diagnosis of CRC at a later age was also refuted. Two other dependencies were refuted, one being the relationship between the mutation and histological grade, and the predilection for metastasis at the peritoneal level. By analyzing the statistical results of other studies, these correlations could also not be demonstrated in samples of sizes similar to that of this study [14,25,26]. This suggests the need for a larger number of cases in order to establish a statistically significant dependency for the cited parameters. Despite the small sample size, the sample was sufficient to suggest a dependency relationship between the BRAF mutation and MSI. This association is likely due to the relationship of BRAF with the high-level CpG island methylation phenotype (CIMP+) and methylation of the MLH1 promoter. This leads to a so-called “sporadic” microsatellite unstable phenotype. Thereupon, it is expected to find MSI more frequently with BRAF-mutated tumors [27]. Consistent with previous reports, the BRAF mutation also occurred more frequently in tumor sites on the right side. CRC is a heterogeneous disease, with the right colon (cecum to transverse colon) and left colon (splenic flexure to rectum) differing in embryologic origin, vascular supply, and physiologic function—all of which influence tumor behavior and prognosis. More importantly, studies have shown that right- and left-sided CRCs have distinct immune landscapes, which play a key role in antitumor immunity. Yet, the reason BRAF mutations occur more frequently on the right side remains unclear and warrants further investigation [28,29].

Cancer treatment studies increasingly rely on molecular epidemiologic data, and this study fills an important gap. The identification of molecular markers can provide insights into the pathogenic process and help optimize personalized cancer therapy. BRAF mutation status is a key component in the management of colorectal cancer, making its prevalence in this poorly studied population particularly important.

There are obviously some limitations to the study. There is a great deal of missing information in the data collection. Also, even though the study sample is one of the largest in the region, the number of patients with BRAF mutation was small and insufficient to establish statistically significant associations between certain variables and the BRAF mutation. To that end, a larger number of tumors with mutated BRAF is needed in order to confirm or deny any association between clinicopathological characteristics to tumors and mutated BRAF profile in a specific population.

## 5. Conclusions

This is the largest study reporting the prevalence of the BRAF mutation in CRC in Lebanon. We found a prevalence of 5.3%, similar to that of neighboring countries, which is significantly lower than that of Western populations. These tumors are often associated with specific patient characteristics and poor prognosis. However, we were not able to establish a significant correlation between BRAF mutation and most of the studied phenotypes due to the limited sample size.

The importance of identifying molecular markers lies in the possibility of understanding the pathogenic process in order to help optimize personalized cancer therapy in the Lebanese population, which remains poorly studied. The treatment of mutated BRAF V600 CRC might still be a challenge, yet new treatment strategies are being developed with promising results.

## Figures and Tables

**Table 1:** Characteristics of the colorectal tumors in the studied population.

Characteristics	Number of Samples ^1^	Result (Percentage)
Age	n = 180	
Average		61.39
Median		63
Gender	n = 190	
Male		101 (53.2%)
Female		89 (46.8%)
Microsatellite	n = 87	
MSS		83 (95.4%)
MSI		4 (4.6%)
Metastases	n = 114	
Peritoneal		70 (61.4%)
Other ^2^		44 (38.6%)
Sidedness	n = 131	
Left		91 (69.5%)
Right		40 (30.5%)
Location	n = 94	
Rectum		41 (43.6%)
Recto-sigmoid		2 (2.1%)
Sigmoid		23 (24.5%)
Left colic flexure		2 (2.1%)
Transverse colon		7 (7.4%)
Right colic flexure		1 (1.1%)
Coecum		17 (18.1%)
Grade	n = 106	
Well-differentiated		9 (8.5%)
Moderately differentiated		88 (83.0%)
Poorly differentiated		9 (8.5%)

^1^ *n* indicates the number of patients with available data for each variant. ^2^ Other refers to non-peritoneal sites (e.g., liver, lung, bone).

**Table 2:** Summary of the relationship between different characteristics of the sample with BRAF status and statistical significance.

	BRAF-Mutated	BRAF Wild-Type	Significant Asymptotic
Gender			*p =* 0.392
Male	4 (4.0%)	97 (96.0%)	
Female	6 (6.7%)	83 (93.3%)	
Microsatellites			*p =* 0.004
MSS	6 (75.0%)	77 (97.5%)	
MSI	2 (25.0%)	2 (2.5%)	
Sidedness			*p =* 0.001
Left	2 (2.2%)	89 (73.0%)	
Right	7 (77.8%)	33 (27.0%)	
Location			*p =* 0.034
Rectum	1 (2.4%)	40 (97.6%)	
Recto-sigmoid	0 (0.0%)	2 (100.0%)	
Sigmoid	0 (0.0%)	23 (100.0%)	
Left colic flexure	1 (33.3%)	2 (66.7%)	
Traverse colon	2 (28.6%)	5 (71.4%)	
Right colic flexure	0 (0.0%)	1 (100.0%)	
Coecum	3 (17.6%)	14 (82.4%)	
Metastases			*p =* 0.707
Peritoneal	5 (55.6%)	65 (61.9%)	
Other	4 (44.4%)	40 (38.1%)	
Histology Grade			*p =* 0.594
Well-differentiated	1 (16.7%)	8 (8.0%)	
Moderately differentiated	5 (83.3%)	83 (83.0%)	
Poorly differentiated	0 (0.0%)	9 (9.0%)	
Age (average)	63.38+/−12.141	61.44+/−13.118	*p =* 0.682

**Table 3:** Sample size and prevalence of BRAF mutation in CRC in various studies from Middle Eastern countries.

Study	Country	Sample Size	Samples with a Mutated BRAF	Prevalence of BRAF
Tony Ibrahim et al., 2018 [13]	Lebanon	31	4	12.9%
Humaid O. Al-Shamsi et al., 2016 [14]	USA *	99	4	4.0%
N. Marchoudi et al., 2013 [15]	Tunisia	92	5	5.4%
Karim Bougatef et al., 2008 [16]	Tunisia	48	4	8.0%
F. Naghibalhossaini et al., 2011 [17]	Iran	110	0	0.0%
M. Molaei et al., 2016 [18]	Iran	85	0	0.0%
Dolatkhah et al., 2015 [19]	Iran	30	0	0.0%
Javadi et al., 2014 [20]	Iran	100	0	0.0%
Alex Vilkin et al., 2009 [21]	Israel	128	24	18.8%
Shaham Beg, MD et al., 2015 [22]	KSA	498	12	2.4%
Ehsan Nazemalhosseini-Mojarad et al., 2020 [23]	Iran	258	15	5.8%
Tamer Garawin et al., 2016 [24]	Algeria, Bahrain, Egypt, Jordan, Kuwait, Lebanon, Libya, Morocco, Oman, Qatar, KSA, UAE	78	3	3.8%
Total		1557	71	4.56%

* The study by Al-Shami et al. took place in the United States, but the population studied is of Middle Eastern origin and has been compared to a Western population.

## Data Availability

Data is contained within the article.

## Abbreviations

The following abbreviations are used in this manuscript:

BRAF	B-Raf proto-oncogene, serine/threonine kinase
CRC	Colorectal cancer
DOAJ	Directory of open access journals
EGFR	Epidermal growth factor receptor
EURO	European Region (World Health Organization)
GTPase	Guanosine triphosphatase
ISO	International Organization for Standardization
KRAS	Kirsten rat sarcoma viral oncogene homolog
LD	Linear dichroism
MDPI	Multidisciplinary Digital Publishing Institute
MEK	Mitogen-activated protein kinase kinase
MSI	Microsatellite instability
MSS	Microsatellite stable
NRAS	Neuroblastoma RAS viral oncogene homolog
P53	Tumor protein p53
PAHO	Pan American Health Organization
PCR	Polymerase chain reaction
PIK3CA	Phosphatidylinositol-4,5-bisphosphate 3-kinase catalytic subunit alpha
PTEN	Phosphatase and tensin homolog
RAS	Rat sarcoma (family of small GTPases)
SPSS	Statistical Package for the Social Sciences
TLA	Three-letter acronym
V600E	Valine-to-glutamic acid substitution at codon 600
WPRO	Western Pacific Region (World Health Organization)
